# Strong interaction between interlayer excitons and correlated electrons in WSe_2_/WS_2_ moiré superlattice

**DOI:** 10.1038/s41467-021-23732-6

**Published:** 2021-06-14

**Authors:** Shengnan Miao, Tianmeng Wang, Xiong Huang, Dongxue Chen, Zhen Lian, Chong Wang, Mark Blei, Takashi Taniguchi, Kenji Watanabe, Sefaattin Tongay, Zenghui Wang, Di Xiao, Yong-Tao Cui, Su-Fei Shi

**Affiliations:** 1 Department of Chemical and Biological Engineering, Rensselaer Polytechnic Institute, Troy, NY USA; 2 Department of Physics and Astronomy, University of California, Riverside, CA USA; 3 Department of Materials Science and Engineering, University of California, Riverside, CA USA; 4 Institute of Fundamental and Frontier Sciences, University of Electronic Science and Technology of China, Chengdu, Sichuan China; 5 Department of Physics, Carnegie Mellon University, Pittsburgh, PA USA; 6 School for Engineering of Matter, Transport and Energy, Arizona State University, Tempe, AZ USA; 7 Research Center for Functional Materials, National Institute for Materials Science, Tsukuba, Japan; 8 International Center for Materials Nanoarchitectonics, National Institute for Materials Science, Tsukuba, Japan; 9 Department of Electrical, Computer and Systems Engineering, Rensselaer Polytechnic Institute, Troy, NY USA

**Keywords:** Two-dimensional materials, Two-dimensional materials, Fluorescence spectroscopy

## Abstract

Heterobilayers of transition metal dichalcogenides (TMDCs) can form a moiré superlattice with flat minibands, which enables strong electron interaction and leads to various fascinating correlated states. These heterobilayers also host interlayer excitons in a type-II band alignment, in which optically excited electrons and holes reside on different layers but remain bound by the Coulomb interaction. Here we explore the unique setting of interlayer excitons interacting with strongly correlated electrons, and we show that the photoluminescence (PL) of interlayer excitons sensitively signals the onset of various correlated insulating states as the band filling is varied. When the system is in one of such states, the PL of interlayer excitons is relatively amplified at increased optical excitation power due to reduced mobility, and the valley polarization of interlayer excitons is enhanced. The moiré superlattice of the TMDC heterobilayer presents an exciting platform to engineer interlayer excitons through the periodic correlated electron states.

## Abstract (web summary)

Heterobilayers of transition metal dichalcogenides host moiré superlattices that give rise to strong electron interactions. Here, the authors study the photoluminescence from interlayer excitons in a WS_2_/WSe_2_ heterobilayer to reveal the onset of various correlated insulating states.

## Introduction

Bilayers of two-dimensional materials with a small twist angle or a lattice mismatch can form moiré superlattices^1–5^. Twisted bilayer graphene at the magic angle possess flat minibands that result in strong electron interactions, enabling rich, correlated states, such as Mott insulators, unconventional superconductivity, and Chern insulators^6–9^. The moiré superlattice of the twisted bilayer transitional metal dichalcogenides (TMDCs) can form flat minibands with less stringent requirements, with no magic angle required. In addition, due to the large effective mass^10–12^, the kinetic energy is further reduced in twisted bilayer TMDCs^13^. The resulting enhanced Coulomb interaction with respect to the kinetic energy leads to strong interactions that give rise to various exotic correlated insulating states^13–21^ with high transition temperatures. Very recently, Mott insulator states at fillings *n* = ±1 (one electron or hole per Moiré cell), generalized Wigner crystal states at filings *n* = ±1/3, ±2/3, and even more exotic insulating states at other fractional fillings have been reported^14–21^. The TMDCs moiré superlattice provides an exciting platform to investigate strongly correlated physics.

Meanwhile, stacking two different TMDC monolayers also enables the possibility for band engineering in the heterobilayer^22,23^. For example, a type-II alignment in the heterobilayer allows the formation of interlayer excitons, in which the optically excited electrons reside in one layer, while the holes reside in the other layer^2,4,24,25^. Due to their long lifetime, valley degree of freedom, and tunability, interlayer excitons are a promising candidate for quantum emitters^3^. Both interlayer excitons and correlated electrons are results of strong interactions, and it is natural to raise the intriguing question of how they interact with each other, which, to our best knowledge, remains an uncharted territory.

In this work, we explore the interaction of interlayer excitons with the correlated states in angle-aligned WS_2_/WSe_2_ moiré superlattice. We find that this interaction significantly modifies the photoluminescence (PL) of the interlayer exciton. Sensitive to the change in dielectric constant and the gap opening at the correlated insulating states, the interlayer exciton PL peak energy unravels the Mott insulator state at *n* = 1, the generalized Wigner crystal states at *n* = 1/3 and 2/3, as well as at *n* = 1/4 and 3/4 with an even longer range interaction, and a stripe phase state at *n* = 1/2. Interestingly, the presence of the correlated states also significantly modifies the PL intensity. At increased optical excitation power, the PL intensity of interlayer excitons brightens up at the correlated electron states, suggesting reduced mobility of interlayer excitons in the moiré cell due to their attractive interaction with the localized correlated electrons. Furthermore, the valley polarization of interlayer excitons is strongly enhanced at certain fillings. Our results inspire engineering interlayer excitons as quantum emitter arrays through moiré potential and long-range interactions, utilizing their interplay with the localized correlated electrons.

## Results

### Correlated states in WSe_2_/WS_2_ heterostructure

**Fig. 1:**
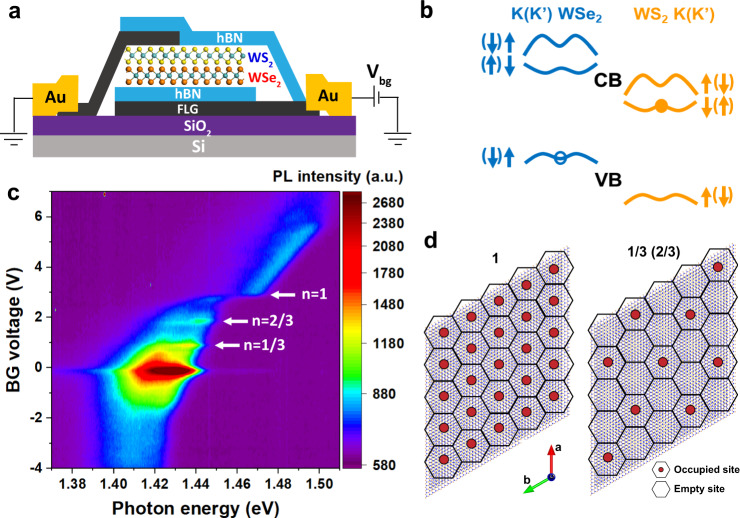
Interlayer exciton PL revealing correlated states in angle-aligned WSe_2_/WS_2_ heterobilayer. **a** Schematic of the hBN encapsulated WS_2_/WSe_2_ heterobilayer. One piece of few-layer graphene (FLG) is used as the contact electrode and another piece is used as the back-gate electrode with the hBN layer working as the gate dielectric. **b** Schematic of the type-II alignment of the WS_2_/WSe_2_ heterobilayer moiré superlattice. **c** Gate-dependent PL spectra of device D1 at 4.2 K, with CW excitation centered at 1.959 eV with an excitation power of 5 µW. **d** Schematics of electron (red dot) configuration for fillings of *n* = 1 and *n* = 1/3 (2/3 and 1/3 are particle-hole symmetric) in the moiré superlattice.

The structure of our angle-aligned WS_2_/WSe_2_ heterobilayer device is schematically shown in Fig. 1a. Due to the ~4% lattice mismatch, the angle-aligned WS_2_/WSe_2_ bilayer would form a triangular moiré superlattice with a period of ~8 nm. The strong moiré coupling^1,5^ will result in mini flatbands^26^, which could host correlated insulating state at half band filling with one carrier localized in one moiré cell (*n* = ±1)^14,15^, or even at various fractional fillings of the moiré unit cell that have been interpreted as generalized Wigner crystal states^14,18,19^ or stripe phases^18–20^. The filling patterns for *n* = 1 and 1/3 are shown schematically in Fig. 1d. These correlated states arise from the strong interaction between carriers, which localizes them in the moiré cells, and the formation of periodic filling pattern minimizes the overall interaction energy. Meanwhile, the energy bands of WS_2_ and WSe_2_ have a type-II alignment in their heterobilayer (Fig. 1b), in which the optically excited electron will reside in monolayer WS_2_ and the corresponding hole will reside in the WSe_2_ layer. Due to the strong Coulomb interaction, the electron–hole pair remain bound as the interlayer exciton.

**Fig. 2:**
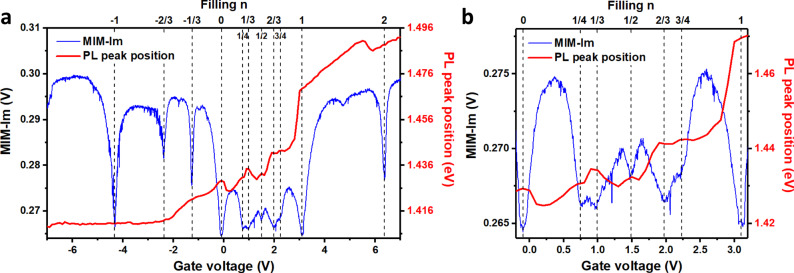
Identification of correlated insulating states at different fillings. **a** PL peak position (red) extracted from Fig. 1c as a function of the gate voltage, correlated with MIM measurements of the local conductivity (blue) taken at 14 K. Dashed lines are fillings corresponding to the insulating states determined from the MIM measurements. **b** Zoom-in of the traces from −0.2 V to 3.2 V in **a**.

In Fig. 1c, we show the color plot of PL intensity as a function of photon energy and gate voltage at 4.2 K, for the interlayer exciton whose emission energy is around 1.4 eV, excited by a continuous wave (CW) laser centered at 1.959 eV with an excitation power of 5 µW. The most noticeable feature is the large energy blueshift (~20 meV) at gate voltage ~2.9 V, when the Mott insulator at *n* = 1 (one electron per moiré cell) is formed, as confirmed by gate-dependent reflectance spectra (see Supplementary Note 2 for details) and local conductivity measurements (Fig. 2a, b). The relatively enhanced PL intensity at the gate voltage of ~0.9 V and ~1.9 V reveals the generalized Wigner crystal states at *n* = 1/3 and 2/3 (Fig. 2a, b), respectively, likely due to the suppressed nonradiative channels at the correlated insulating states. A closer look of Fig. 1c could even resolve the states corresponding to *n* = 1/4 and 3/4 (see Supplementary Fig. 2).

### Microwave impedance microscopy

From Fig. 1c, we extract the PL peak energy position as a function of gate voltage and correlate it with the local conductivity measured through microwave impedance microscopy (MIM) on the same device D1 (see Fig. 2a). The various dips in the MIM signal indicate the appearance of insulating states whose filling values can be determined by matching its pattern with our prior report^19^. From the MIM data, we identify insulating states at fillings *n* = 1/4, 1/3, 1/2, 2/3, 3/4, 1, and 2 on the electron side, and *n* = −1/3, −2/3, and −1 on the hole side. Comparing the MIM with the PL peak position data, we find that most of these fillings also appear in the PL data. The most pronounced ones are the fillings on the electron side, which show blue shifts in the PL peak position. These observed filling values are consistent with recent reports^18,19^. Among them, the *n* = 2 corresponds to the complete filling of the first minibands and the *n* = ±1 correspond to the Mott insulator states at half filling of these minibands^14,15,21^. The *n* = ±1/3, ±2/3, +1/4, and +3/4 correspond to generalized Wigner crystals with a triangular lattice^18,19^. The *n* = 1/2 corresponds to a stripe phase^18–20^.

The sensitive readout of the correlated insulating states through the interlayer exciton PL energy can be understood by the larger spatial extent of the interlayer exciton, which renders it more sensitive to the dielectric environment than the intralayer exciton. Similar ideas has been utilized to use the larger 2*s* exciton to sensitively probe the correlated insulating states^18^. In addition, the background free nature makes the PL spectra a sensitive probe. At the correlated insulating states, electron interaction results in an energy gap and localizes the electrons, which subsequently modifies the dielectric constant. As a result, both the energy gap seen by the interlayer excitons and their binding energies can be modified, leading to the change in the emission energy, observed as the blueshift of the PL peak at the filling of the correlated states. This picture is consistent with our observation of the larger PL blueshift at *n* = 1, as the Mott insulator state has a larger gap (~10 meV) compared with other fractional fillings (~2–3 meV) according to previous reports^14,15,18,19^.

### Power-dependent PL spectra of interlayer excitons

**Fig. 3:**
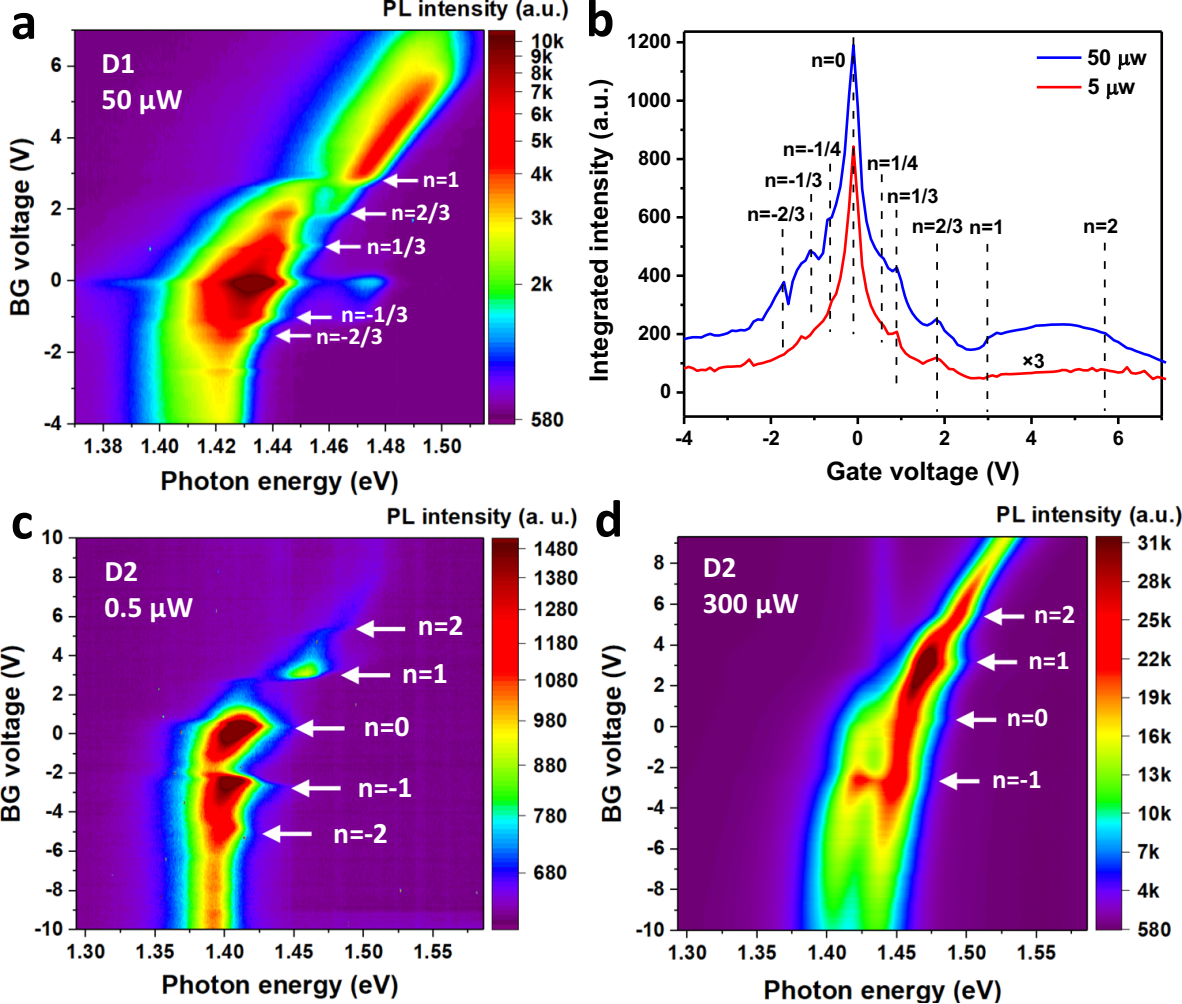
PL of interlayer excitons under increased optical excitation power. **a** Gate-dependent PL spectra of device D1 with the optical excitation power of 50 µW and photon energy centered at 1.959 eV. **b** Integrated interlayer exciton PL intensity as a function of gate voltage for device D1 with 50 µW (blue) and 5 µW (red) optical excitation power. Dashed lines are fillings determined from the insulating states from the MIM measurements. **c**, **d** Gate-dependent PL spectra for device D2 under the optical excitation power of 0.5 and 300 µW. All data are taken at 4.2 K.

The interaction among the interlayer excitons and that between the excitons and the underlying electron solid can be further probed by increasing the interlayer exciton density. Figure 3a shows the color plot of the gate-dependent PL spectra of device D1 at an optical excitation power ten times (50 µW) as much as that used in Fig. 1c (5 µW). Compared with Fig. 1c, the higher excitation power data now reveals the states at *n* = −1/3 and −2/3, which are previously missing in the lower excitation power data. Detailed analysis of the integrated PL intensity as a function of the gate voltage shown in Fig. 3b further reveals the state corresponding to *n* = −1/4. We perform even more drastic excitation power dependence on another device D2, in which we increase the excitation power from 0.5 µW (Fig. 3c) to 300 µW (Fig. 3d). It is obvious that despite the increase of optical excitation by nearly three orders of magnitude, the Mott insulator states at *n* = ±1 remain intact. This observation is consistent with the picture that the excitons do not destroy the periodic electron solid^4,27^. It is worth noting that the asymmetric PL peak shift as a function of the gate voltage for the *p*- and *n*-doping sides is due to the difference in the Stark effect related to our specific device structure, which is back gated with the WSe_2_ layer closer to the back-gate electrode. Owing to the type-II alignment between WSe_2_ and WS_2_, the gate voltage induced holes in the *p*-doping side will occupy the WSe_2_ layer, screening the electrical field between the WSe_2_ and WS_2_ layer, and reducing the Stark effect. A detailed explanation can be found in Supplementary Note 5.

One other striking observation is that, in contrast to the intense PL intensity at the charge neutral region (*n* = 0) under the low optical excitation power (Fig. 3c), the PL intensity at the *n* = 1 state is more strongly enhanced under high excitation power (Fig. 3d). The time-resolved PL spectra (TRPL) shows a lifetime of 6.5 ns for the *n* = 1 state (PL peak at ~1.47 eV) and 5.1 ns for the *n* = 0 state (PL peak at ~1.45 eV) (see Supplementary Note 3 for details). This might arise from the mobility reduction of interlayer excitons at the correlated state at *n* = 1, in which electrons are localized in the periodic moiré cells. Interlayer excitons can be polarized by the localized charges, inducing an attractive force between the correlated electrons and interlayer excitons that reduces the interlayer exciton mobility compared to the charge neutral scenario (*n* = 0). As a result, the chance of nonradiative recombination is reduced, consistent with the TRPL data. It has been shown that the moiré potential can trap interlayer excitons^2,4,28,29^. Our results naturally raise the question of possible localization of interlayer excitons through the additional attraction by the periodic correlated electron solid, which we will explore in future studies.

### Gate-dependent valley polarization

**Fig. 4:**
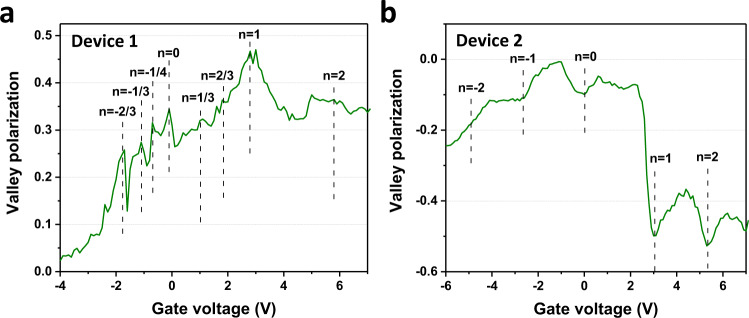
Valley polarization of interlayer excitons in the moiré superlattice. **a**, **b** Valley polarization for the interlayer excitons in the devices D1 (0° twist angle) and D2 (60° twist angle). The dashed lines are the fillings determined from the MIM measurements. Data are taken at 4.2 K.

Interlayer excitons also possess valley degree of freedom^30^, which can be probed through helicity-resolved PL spectroscopy measurements. Here we excite both the devices D1 and D2 using the right circularly polarized light (*σ*^+^) and detect the interlayer exciton PL of the same (*σ*^+^) or opposite (*σ*^−^) helicity. The valley polarization $P$ is calculated by the expression $P=\frac{I\left({\sigma }^{+}\right)-I({\sigma }^{-})}{I\left({\sigma }^{+}\right)+I({\sigma }^{-})}$, where $I\left({\sigma }^{+}\right)$ and I$\left({\sigma }^{-}\right)$ are interlayer exciton PL intensities with ${{\rm{\sigma }}}^{+}$ and ${{\rm{\sigma }}}^{-}$ helicity, respectively. The valley polarizations as a function of gate voltage for the devices D1 and D2 at 4.2 K are shown in Fig. 4a, b, respectively. The overall valley polarizations for these two devices have different signs due to the different alignment angles (0° for D1 and 60° for D2), and the sign of the valley polarization is consistent with the interlayer exciton at the atomic registry site of ${R}_{h}^{X}$ and ${H}_{h}^{h}$ for the devices D1 and D2, respectively^31^. It is noticeable that the correlated states at both integer and fractional fillings are often associated with an enhanced valley polarization, and the enhancement is most drastic at the fillings of *n* = 1 and 2 for device D2. This valley polarization enhancement might originate from the decreased valley scatterings due to the interaction between interlayer excitons and correlated electrons. One major valley depolarization mechanism is through Maialle–Silva–Sham mechanism^32,33^, which annihilates one exciton in one valley and creates another in the opposite valley. However, when interlayer excitons are attracted by the localized correlated electrons, this probability will likely be significantly suppressed. The correlated electrons, therefore, also provide a tunable platform to manipulate the valleyspin of the interlayer excitons.

Researchers have been inspired to utilize the moiré potential to control interlayer excitons to form an array of quantum emitters. Here we show that the long-range interaction of the electrons in the moiré superlattice can be further exploited for this purpose. The interaction between correlated electrons and interlayer excitons not only allows us to reveal the correlated states at different fillings through the PL spectra, more excitingly, it can be further utilized to control the mobility and valley polarization of the interlayer excitons. Our results, therefore, shed light on how to engineer the interlayer excitons in the TMDCs heterobilayer moiré superlattice for quantum optics and optoelectronics.

## Methods

### Heterostructure device fabrication

We use a layer-by-layer pickup method to fabricate the WS_2_/WSe_2_ heterobilayer device^34^. We first exfoliate monolayer WS_2_, monolayer WSe_2_, few-layer graphene and thin hBN flakes on Si substrate with 285 nm thermal oxide, respectively. We further confirm the crystal orientation of monolayer WSe_2_ and WS_2_ through second harmonic generation (SHG) measurements (see Supplementary Note 1 for details). We then mount the SiO_2_/Si chip on a rotational stage and use hBN as a stamp to pick up each layer sequentially. The alignment of the monolayer WS_2_ and WSe_2_ are controlled by fine adjusting the angle of the rotational stage (accuracy of 0.02°) under the microscope, ensuring a near-zero twist angle between the two flakes. The final device is annealed at 140 °C for 6 h in a vacuum chamber. The pre-patterned Au contact electrodes are fabricated through standard electron-beam lithography followed by ebeam evaporation and lift-off processes.

### Optical characterization

The optical measurements are taken with a home-built confocal microscope with spectroscopy capabilities in a liquid helium-controlled optical cryostat. The PL spectra are taken with a CW laser excitation. The reflectance spectra are taken with a supercontinuum white laser source (Fianium). The SHG measurements are taken with a pulsed laser with the width ~160 fs (Ti : Sapphire, Coherent Chameleon).

### MIM measurements

The MIM measurement is performed on a home-built cryogenic scanning probe microscope platform. A small microwave excitation of ~0.1 µW at a fixed frequency around 10 GHz is delivered to a chemically etched tungsten tip mounted on a quartz tuning fork^35^. The reflected signal is analyzed to extract the demodulated output channels, MIM-Im and MIM-Re, which are proportional to the imaginary and real parts of the admittance between the tip and the sample, respectively. The MIM-Im part characterizes the screening of the tip electric field by the sample and changes monotonically with the sample’s local conductivity. Therefore, the dips in the MIM-Im curve plotted in Fig. 2 indicate the appearance of insulating states.

## Supplementary information

Supplementary Information

## Data Availability

The data that support the findings of this study are available from the corresponding author upon reasonable request.

## References

[1] Jin C (2019). Observation of moiré excitons in WSe_2_/WS_2_ heterostructure superlattices. Nature.

[2] Seyler KL (2019). Signatures of moiré-trapped valley excitons in MoSe_2_/WSe_2_ heterobilayers. Nature.

[3] Yu H, Liu G, Tang J, Xu X, Yao W (2017). Moiré excitons: from programmable quantum emitter arrays to spin-orbit – coupled artificial lattices. Sci. Adv..

[4] Tran K (2019). Evidence for moiré excitons in van der Waals heterostructures. Nature.

[5] Alexeev EM (2019). Resonantly hybridized excitons in moiré superlattices in van der Waals heterostructures. Nature.

[6] Bistritzer R, MacDonald AH (2011). Moiré bands in twisted double-layer graphene. Proc. Natl Acad. Sci. USA.

[7] Cao Y (2018). Correlated insulator behaviour at half-filling in magic-angle graphene superlattices. Nature.

[8] Cao Y (2018). Unconventional superconductivity in magic-angle graphene superlattices. Nature.

[9] Andrei EY, MacDonald AH (2020). Graphene bilayers with a twist. Nat. Mater..

[10] Kono J (2018). Magnetooptics of exciton Rydberg states in a monolayer semiconductor. Phys. Rev. Lett..

[11] Wang T (2020). Giant valley-polarized Rydberg excitons in monolayer WSe_2_ revealed by magneto-photocurrent spectroscopy. Nano Lett..

[12] Li Z (2020). Phonon-exciton Interactions in WSe_2_ under a quantizing magnetic field. Nat. Commun..

[13] Wu F, Lovorn T, Tutuc E, MacDonald AH (2018). Hubbard model physics in transition metal dichalcogenide moiré bands. Phys. Rev. Lett..

[14] Regan EC (2020). Mott and generalized Wigner crystal states in WSe_2_/WS_2_ moiré superlattices. Nature.

[15] Tang Y (2020). Simulation of Hubbard model physics in WSe_2_/WS_2_ moiré superlattices. Nature.

[16] Shimazaki Y (2020). Strongly correlated electrons and hybrid excitons in a moiré heterostructure. Nature.

[17] Wang L (2020). Correlated electronic phases in twisted bilayer transition metal dichalcogenides. Nat. Mater..

[18] Xu Y (2020). Correlated insulating states at fractional fillings of moiré superlattices. Nature.

[19] Huang, X. et al. Correlated insulating states at fractional fillings of the WS_2_/WSe_2_ moiré lattice. *Nat. Phys.*10.1038/s41567-021-01171-w (2021).

[20] Jin, C. et al. Stripe phases in WSe_2_/WS_2_ moiré superlattices. *Nat. Mater.*10.1038/s41563-021-00959-8 (2021). 10.1038/s41563-021-00959-8 33767398

[21] Chu Z (2020). Nanoscale conductivity imaging of correlated electronic states in WSe_2_/WS_2_ moiré superlattices. Phys. Rev. Lett..

[22] Ozcelik, V. O., Azadani, J. G., Yang, C., Koester, S. J. & Low, T. Band alignment of two-dimensional semiconductors for designing heterostructures with momentum space matching. *Phys. Rev. B Condens. Matter Mater. Phys*. **94**, 035125 (2016).

[23] Chiu MH (2015). Determination of band alignment in the single-layer MoS_2_/WSe_2_ heterojunction. Nat. Commun..

[24] Rivera P (2015). Observation of long-lived interlayer excitons in monolayer MoSe_2_/WSe_2_ heterostructures. Nat. Commun..

[25] Wang T (2020). Giant valley-Zeeman splitting from spin-singlet and spin-triplet interlayer excitons in WSe_2_/MoSe_2_ heterostructure. Nano Lett..

[26] Zhang Z (2020). Flat bands in twisted bilayer transition metal dichalcogenides. Nat. Phys..

[27] Smoleński, T. et al. Observation of Wigner crystal of electrons in a monolayer semiconductor. Preprint at https://arXiv:2010.03078 (2020). 10.1038/s41586-021-03590-4 34194018

[28] Choi J (2020). Moiré potential impedes interlayer exciton diffusion in van der Waals heterostructures. Sci. Adv..

[29] Yuan L (2020). Twist-angle-dependent interlayer exciton diffusion in WS_2_/WSe_2_ heterobilayers. Nat. Mater..

[30] Xu X, Yao W, Xiao D, Heinz TF (2014). Spin and pseudospins in layered transition metal dichalcogenides. Nat. Phys..

[31] Yu H, Liu G-B, Yao W (2018). Brightened spin-triplet interlayer excitons and optical selection rules in van der Waals heterobilayers. 2D Mater..

[32] Chen SY (2018). Superior valley polarization and coherence of 2s excitons in monolayer WSe_2_. Phys. Rev. Lett..

[33] Maialle MZ, de Andrada e Silva EA, Sham LJ (1993). Exciton spin dynamics in quantum wells. Phys. Rev. B.

[34] Wang L (2013). One-dimensional electrical contact to a two-dimensional material. Science.

[35] Cui Y-T, Ma EY, Shen Z-X (2016). Quartz tuning fork based microwave impedance microscopy. Rev. Sci. Instrum..

