# Supplementary material for: Strong interaction between interlayer excitons and correlated electrons in WSe2/WS2 moiré superlattice
Source: Nat Commun. 2021 Jun 14;12:3608. doi: 10.1038/s41467-021-23732-6 (PMC8203657; doi:10.1038/s41467-021-23732-6)
Supplement: Supplementary file 1 — Supplementary Information [file 41467_2021_23732_MOESM1_ESM.pdf]

Supplementary Information

Strong Interaction Between Interlayer Excitons and Correlated Electrons in WSe_2/WS_2 Moiré Superlattice

Shengnan Miao[1#], Tianmeng Wang[1#], Xiong Huang[2,3#], Dongxue Chen[1,4#], Zhen Lian[1], Chong Wang[5], Mark Blei[6], Takashi Taniguchi[7], Kenji Watanabe[8], Sefaattin Tongay[6], Zenghui Wang[4*], Di Xiao[5], Yong-Tao Cui[2*], Su-Fei Shi[1,9*]

1. Department of Chemical and Biological Engineering, Rensselaer Polytechnic Institute, Troy, NY 12180, USA
2. Department of Physics and Astronomy, University of California, Riverside, California, 92521, USA
3. Department of Materials Science and Engineering, University of California, Riverside, California, 92521, USA
4. Institute of Fundamental and Frontier Sciences, University of Electronic Science and Technology of China, Chengdu, Sichuan, China.
5. Department of Physics, Carnegie Mellon University, Pittsburgh, PA 15213, USA
6. School for Engineering of Matter, Transport and Energy, Arizona State University, Tempe, AZ 85287, USA
7. Research Center for Functional Materials, National Institute for Materials Science, 1-1 Namiki, Tsukuba 305-0044, Japan
8. International Center for Materials Nanoarchitectonics, National Institute for Materials Science, 1-1 Namiki, Tsukuba 305-0044, Japan
9. Department of Electrical, Computer & Systems Engineering, Rensselaer Polytechnic Institute, Troy, NY 12180, USA

[#] These authors contributed equally to this work
[*] Corresponding authors:
shis2@rpi.edu, yongtao.cui@ucr.edu, zenghui.wang@uestc.edu.cn

Supplementary Note 1. Device information

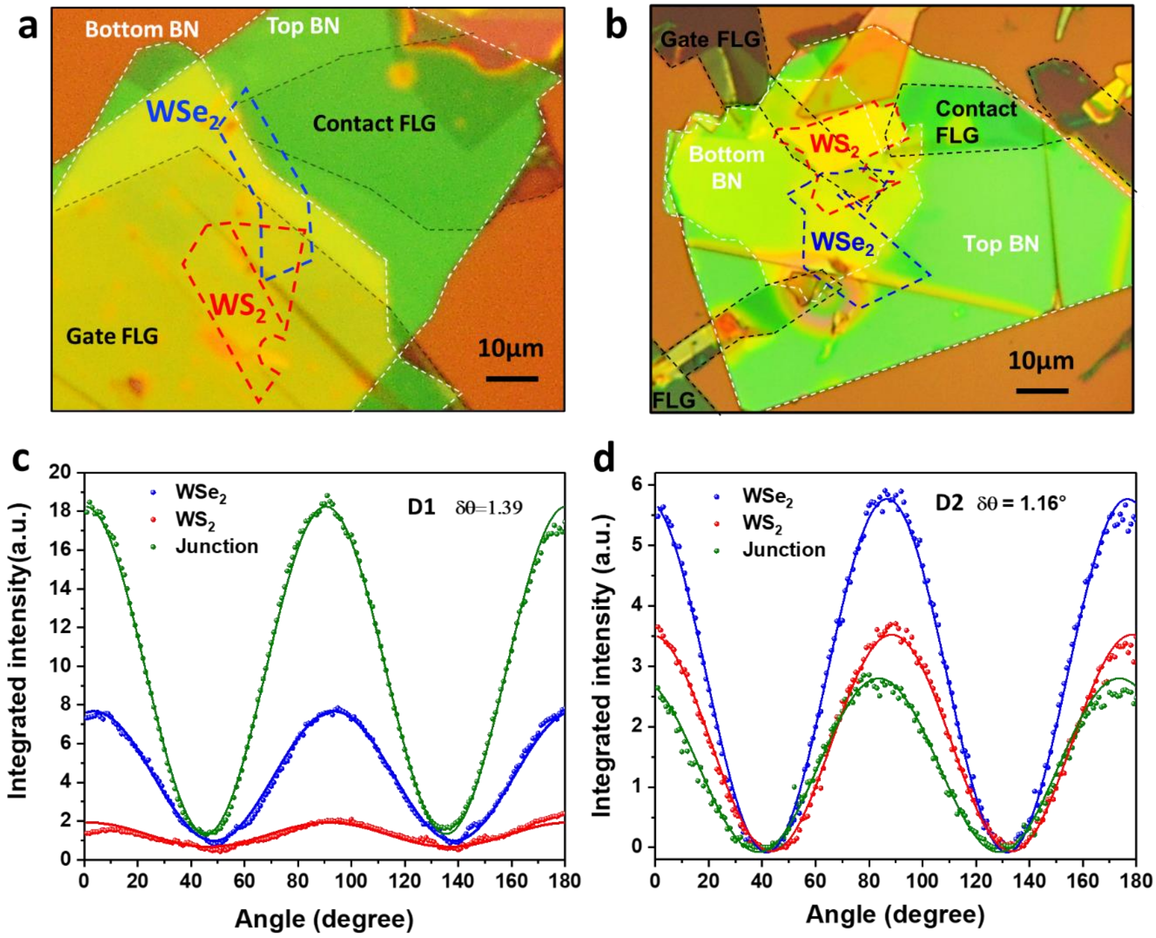

Supplementary Figure 1. Optical microscope images of devices (a) D1 and (b) D2 with different flakes outlined. (c) and (d) are the angular dependence of the SHG signal in device D1 and device D2. Here $\delta\theta$ means the misalignment of the twist angle for device D1 (0-degree twisted) and device D2 (60-degree twisted).

Supplementary Note 2. Additional optical information for device D1 and device D2

The zoom-in of the gate dependent PL spectra shown as the Fig. 1c of the main text is shown in Supplementary Fig. 2, and a close look can even resolve the n=1/4 and 3/4 states directly.

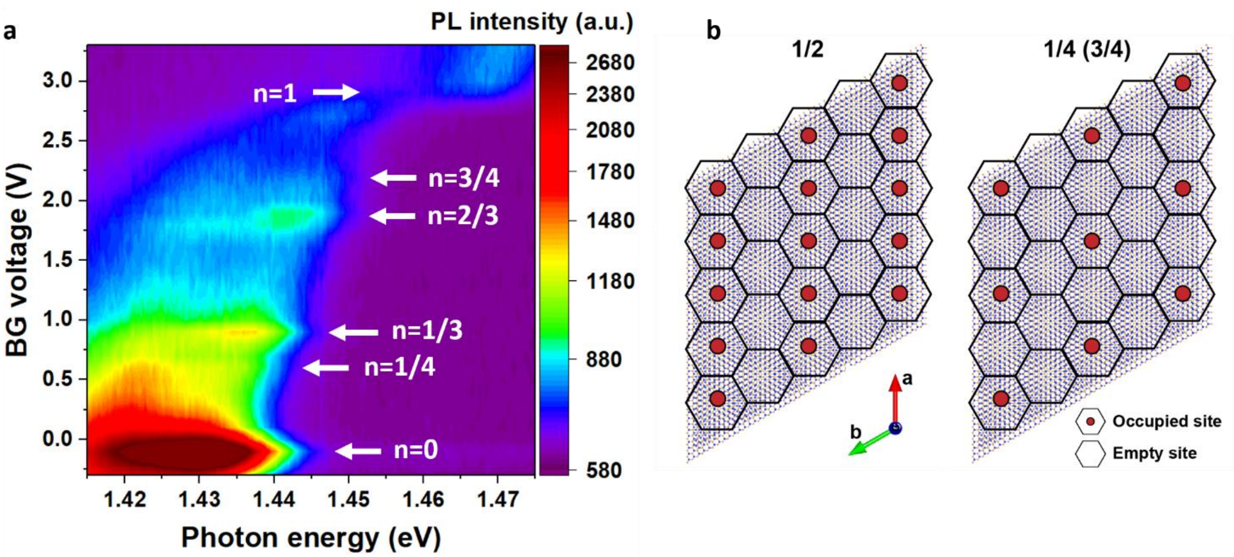

Supplementary Figure 2. (a) Zoom-in gate-dependent PL spectra from -0.3 V to 3.3 V of device D1 at 4.2 K, with CW excitation centered at 1.959 eV and an excitation power of 5 μW. (b) Schematic representations of the correlated electron states at the filling of 1/2 and 1/4 (3/4).

We also perform the optical reflectance measurement on device D1 and D2 at 4.2 K, using a supercontinuum white laser source. Overall, moiré excitons of WSe_2 can be resolved at n=0 (charge-neutral) for both devices and the correlated states at n=±1 are evident for both devices. The difference in gate dependent reflectance spectra for D1 and D2 are from the different alignment angle (0 degree for D1 and 60 degree for D2).

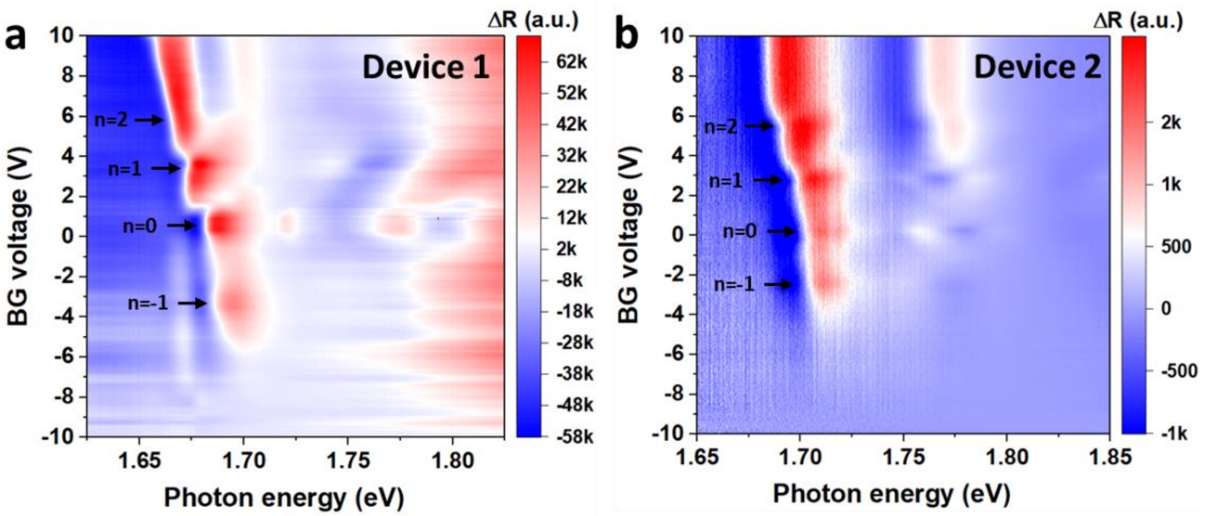

Supplementary Figure 3. Gate-dependent reflection spectra of devices (a) D1 and (b) D2 at 4.2 K.

Supplementary Note 3. Time-resolved PL spectra of n=1 state and n=0 state

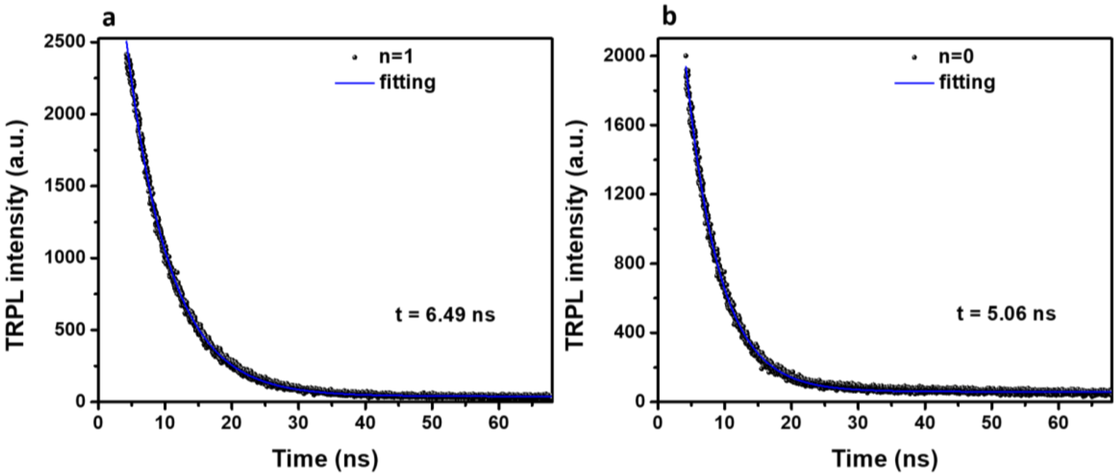

Supplementary Figure 4. TRPL spectra for the PL centered at ~1.45 eV (n=0) and ~1.47 eV (n=1) for D2 at 4.2 K, with 1 mW pulsed laser (Fianium) excitation centered at 1.908 eV

Supplementary Note 4. Helicity-resolved PL spectra of device D1 and device D2

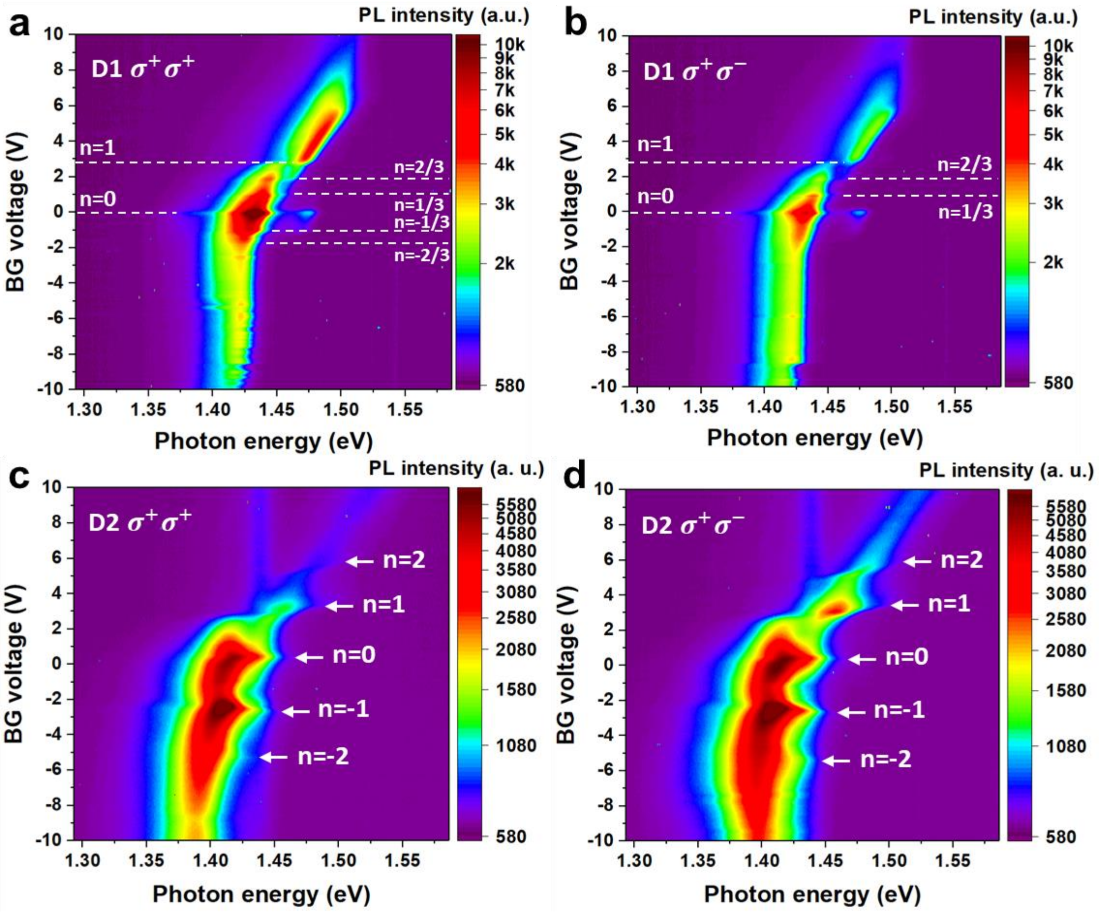

Supplementary Figure 5. (a) and (b) are gate dependent PL spectra of device D1 with the optical excitation power of 50 µW and photon energy centered at 1.959 eV, using right circularly polarized light (σ+) as the excitation and detect the interlayer exciton PL of the same (σ+) or opposite (σ-) helicity. (c) and (d) are gate dependent PL spectra of device D2 with the optical excitation power of 5 µW and photon energy centered at 1.959 eV, using right circularly polarized light (σ+) as the excitation and detect the interlayer exciton PL of the same (σ+) or opposite (σ-) helicity.

Supplementary Note 5. DC Stark shift of interlayer exciton

The apparent asymmetry of the PL peak position for electron and hole doping regions in Fig. 1c and Fig. 2a in the main text is due to the difference in the Stark effect in the p and n-doping regions, determined by the stacking order of the WSe_2/WS_2 bilayer.

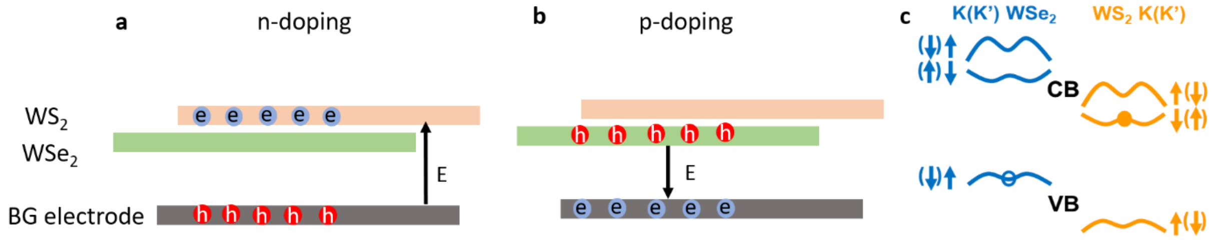

Supplementary Figure 6. Schematic representation of the back gated WSe_2/WS_2 device.

As shown in Supplementary Fig. 6c, the WSe_2/WS_2 heterostructure forms a type II alignment, and the electron tends to reside in the WS_2 layer and the hole tends to reside in the WSe_2 layer. When the heterobilayer is n-doped, electrons accumulate in the WS_2 layer (Supplementary Fig. 6a). As the WSe_2 layer is intrinsic, the screening of electric field by the WSe_2 layer is negligible. When the heterobilayer is p-doped, holes accumulate in the WSe_2 layer (Supplementary Fig. 6b), which significantly screens the electric field. As a result, the electrical field between the WSe_2 and WS_2 layer is negligible, much smaller than that of the n-doing region. Therefore, the blue shift due to the Stark shift is pronounced in the n-doping region, but much reduced in the p-doping region.

Supplementary Note 6. MIM data from device D2

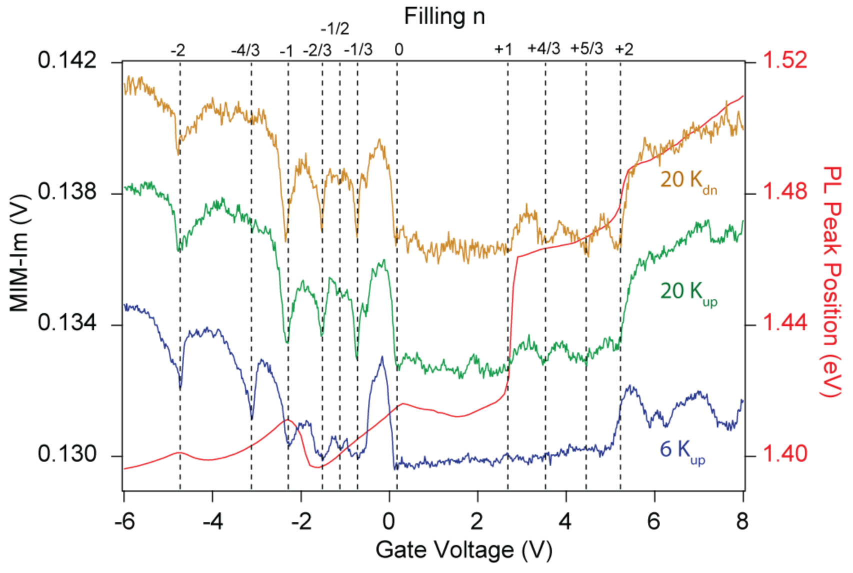

Supplementary Figure 7. (a) PL peak position (red) extracted from Fig. 3c in the main text as a function of the gate voltage, correlated with MIM measurements of the local conductivity (blue) taken at 6 K. Dashed lines are fillings corresponding to the insulating states determined from the MIM measurements.

Supplementary Note 7. Calibration of the filling

First, we identify the major insulating states at n=±1/3, ±2/3, and ±1, through temperature dependent MIM measurements, which is shown in Supplementary Fig. 8. The 3-denominator states disappear around 30 K while n=±1 fillings persist up to much higher temperatures. Then by assuming a linear relation between gate voltage and carrier density, we can determine the filling values for all other fine features.

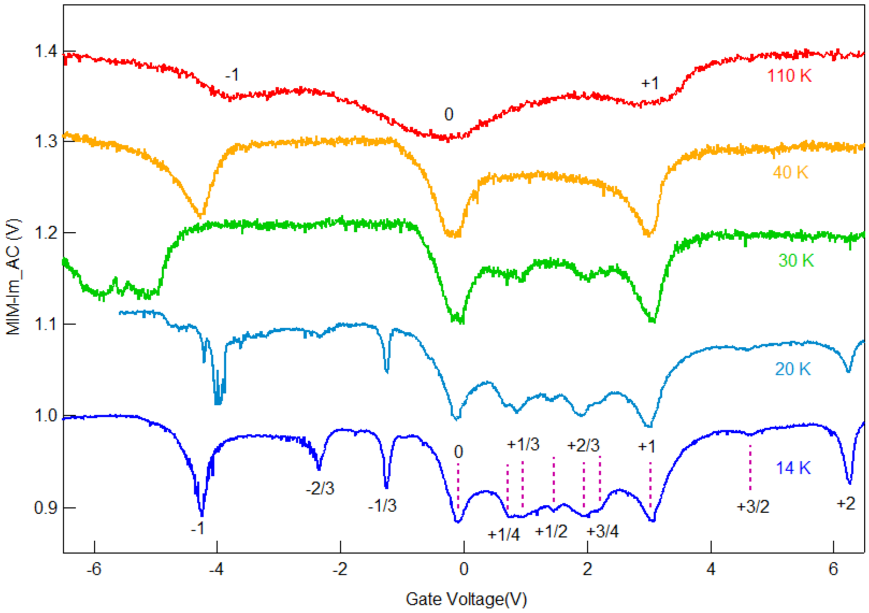

Supplementary Figure 8. MIM-Im versus gate voltage curves taken at temperatures from 14 K to 110 K.
